# Platelet activation via dynamic conformational changes of von Willebrand factor under shear

**DOI:** 10.1371/journal.pone.0234501

**Published:** 2020-06-11

**Authors:** Denis M. Pushin, Tatiana Y. Salikhova, Ksenia E. Zlobina, Georgy Th. Guria

**Affiliations:** 1 National Research Center for Hematology, Moscow, Russia; 2 Moscow Institute of Physics and Technology, Dolgoprudny, Russia; Universiteit Gent, BELGIUM

## Abstract

Shear-induced conformational changes of von Willebrand factor (VWF) play an important role in platelet activation. A novel approach describing VWF unfolding on the platelet surface under dynamic shear stress is proposed. Cumulative effect of dynamic shear on platelet activation via conformational changes of VWF is analysed. The critical condition of shear-induced platelet activation is formulated. The explicit expression for the threshold value of cumulative shear stress as a function of VWF multimer size is derived. The results open novel prospects for pharmacological regulation of shear-induced platelet activation through control of VWF multimers size distribution.

## Introduction

It is known that high shear stresses induce platelet activation and subsequent aggregation [1–4]. This process is premised on the conformational unfolding of von Willebrand factor (VWF) molecules. Under certain shear flows, VWF multimers unfold, exposing more A1 domains capable of binding platelet receptors GPIb [5–9]. The initial activation of platelet signal cascades is triggered by the simultaneous coating of several GPIb receptors with A1 domains of a VWF molecule [10–12]. Multivalent binding of VWF molecules makes possible subsequent platelet aggregation. According to the current viewpoint, shear-induced platelet aggregation (SIPA) plays an important role in the development of myocardial infarction, stroke and some other diseases [13–16]. For that reason, SIPA has been intensively investigated *in vivo* and *in vitro* [17–19].

Under spatially uniform hydrodynamic conditions, *in vitro* platelet activation takes place at steady shear stress exceeding 80 (*dyn*/*cm*^2^) [20–22]. In contrast, under unsteady conditions, particularly when blood moves in stenotic vessels, platelets can undergo high shear stress for only short time intervals [23]. Under such conditions, shear stress must act on platelets with VWF during a time interval sufficient for initial platelet activation. For this reason, it has been suggested that platelets become activated if the cumulative shear stress (*CSS*) is greater than a certain critical value (*CSS*_0_) [24–27]:

$$CSS\equiv \int_{t_{in}}^{t_{out}}\tau \left(t\right)dt\geq CSS_{0}$$ (1)

where *τ*(*t*) is the shear stress to which platelets are exposed under unsteady shear conditions at time *t*; *t*_*in*_ and *t*_*out*_ correspond to the time points when platelets move in and out of the high shear stress zone, respectively.

Data on the value of the threshold cumulative shear stress *CSS*_0_ is highly controversial [28–34]. To the best of our knowledge, there has been no discussion of how the value of *CSS*_0_ can depend on physicochemical properties of blood, including composition, degree of biomacromolecule polymerization, etc.

In the current work, we propose an approach that allows us to analytically derive an expression for the condition of platelet activation under unsteady shear stress. The approach is based on the idea that under unsteady flow, the conformation of VWF molecules grafted on platelet surfaces can be dynamically changed. As a result of VWF unfolding, the efficient connection with multiple GPIb receptors on the platelet surface is increased. When the degree of connection is increased above a critical value, the platelet is assumed to be primed for activation. Under these assumptions, it was shown that platelet activation should truly take place if cumulative shear stress exceeds the definite threshold level. As a result of the proposed approach the dependence of the threshold value of cumulative shear stress as a function of the multimeric size of VWF molecules was derived.

## Materials and methods

### Platelet activation in steady and unsteady shear flow

**Fig 1:**
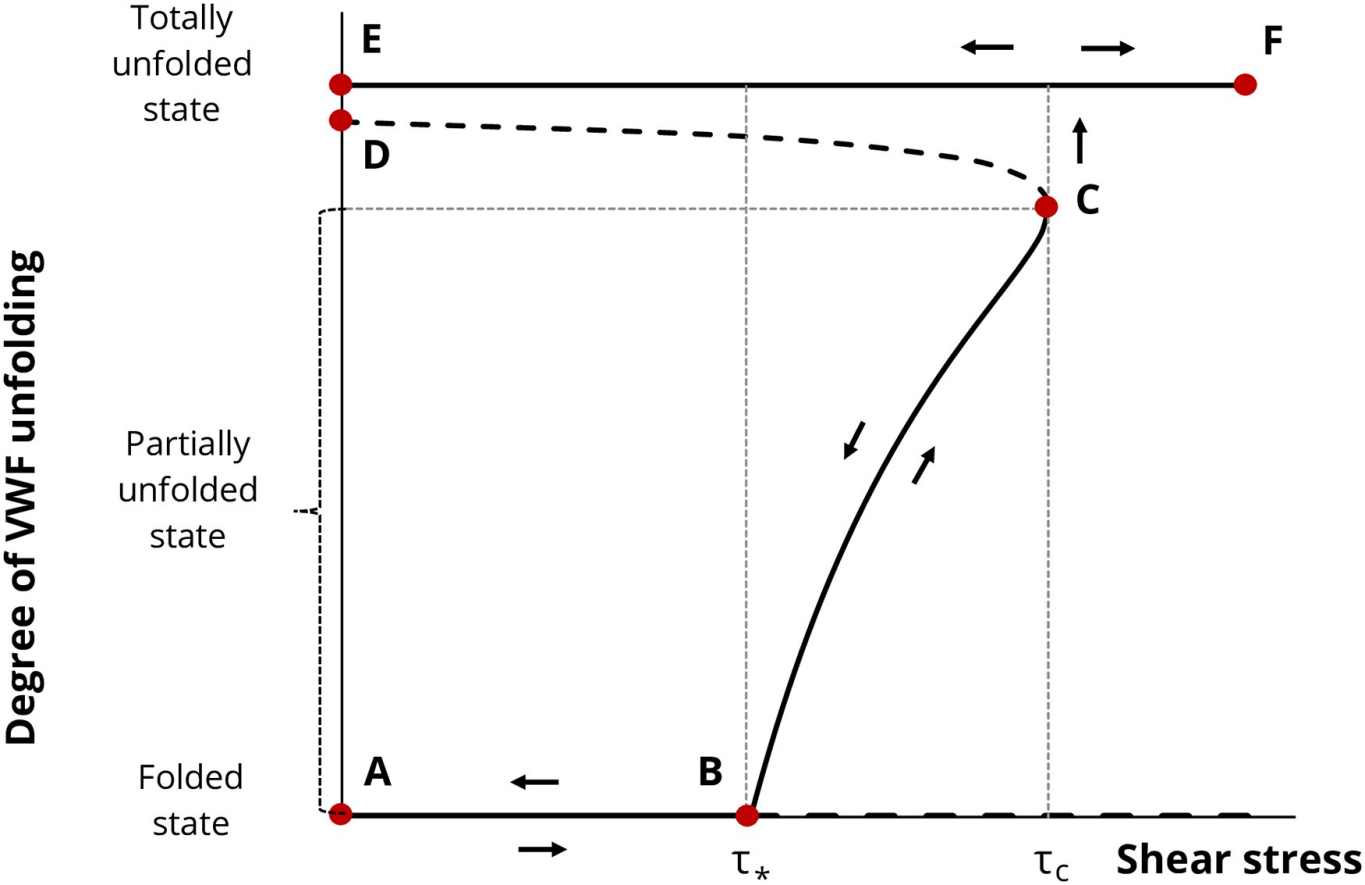
Bifurcation diagram of VWF conformational stability at different shear stresses. Bold lines display branches corresponding to stable steady states and dashed lines display branches corresponding to unstable steady states. τ_*_ is a bifurcation value of shear stress. The value *τ*_*c*_ is a value of shear stress under which VWF molecules must be unwound to their full length. Degree of unfolding (*u*) denotes a ratio of the tail length of the VWF molecule to the VWF contour length (*u* = *x*/*L*). Adapted from [35].

The unfolding of VWF macromolecules on the surface of platelets under steady shear stress was analysed previously [35]. It was established that the conformational state of VWF molecules adsorbed on the surface of platelets depends on the shear stress value. A bifurcation diagram displaying the dependence of the unfolding degree of VWF molecules on shear stress is shown in Fig 1.

The diagram shows that at low shear stress, *τ* < τ_*_, there are two stable states of VWF molecules (folded and totally unfolded). If a VWF molecule is initially in globular form on the platelet surface when the shear stress starts to exceed the critical value τ_*_, partial unfolding of the molecule should begin (branch BC). A further increase in shear stress leads to a second critical value *τ*_*c*_, at which the partially unfolded state disappears as a result of fold catastrophe (point C). The system inevitably evolves to a completely unfolded state. From the bifurcation diagram (Fig 1), it is also clear that an unfolded state exists and is stable with respect to finite amplitude perturbation at all shear stress values either greater or less than *τ*_*c*_ (branch EF).

It also follows from the diagram that any partially unfolded state should return to the folded state during the gradual decline in shear stress *τ* (movement along the BC branch from point C to point B). This means that platelet activation by partially unfolded VWF molecules can take place only in the cases when the duration of overcritical shear stress is permanent or rather long [35].

Unlike partially unfolded states, VWF molecules in totally unfolded states remain resistant to any quasistatic changes in shear stress values. This means that if the VWF molecule was ever completely unfolded, it would remain in the completely unfolded state upon further increasing or decreasing the shear stress. In particular, if VWF molecules become completely unfolded as a result of platelets passing through stenotic regions of blood vessels containing high shear stress zones, molecules will continue to circulate in the blood in that completely unfolded state. Therefore, the exposure time of platelet GPIb receptors to VWF molecule action will not be limited by the time passing through the stenotic region.

In this paper, we investigate the conditions of VWF molecule total unfolding under the dynamic action of unsteady shear stress.

### VWF dynamics under unsteady shear stress

**Fig 2:**
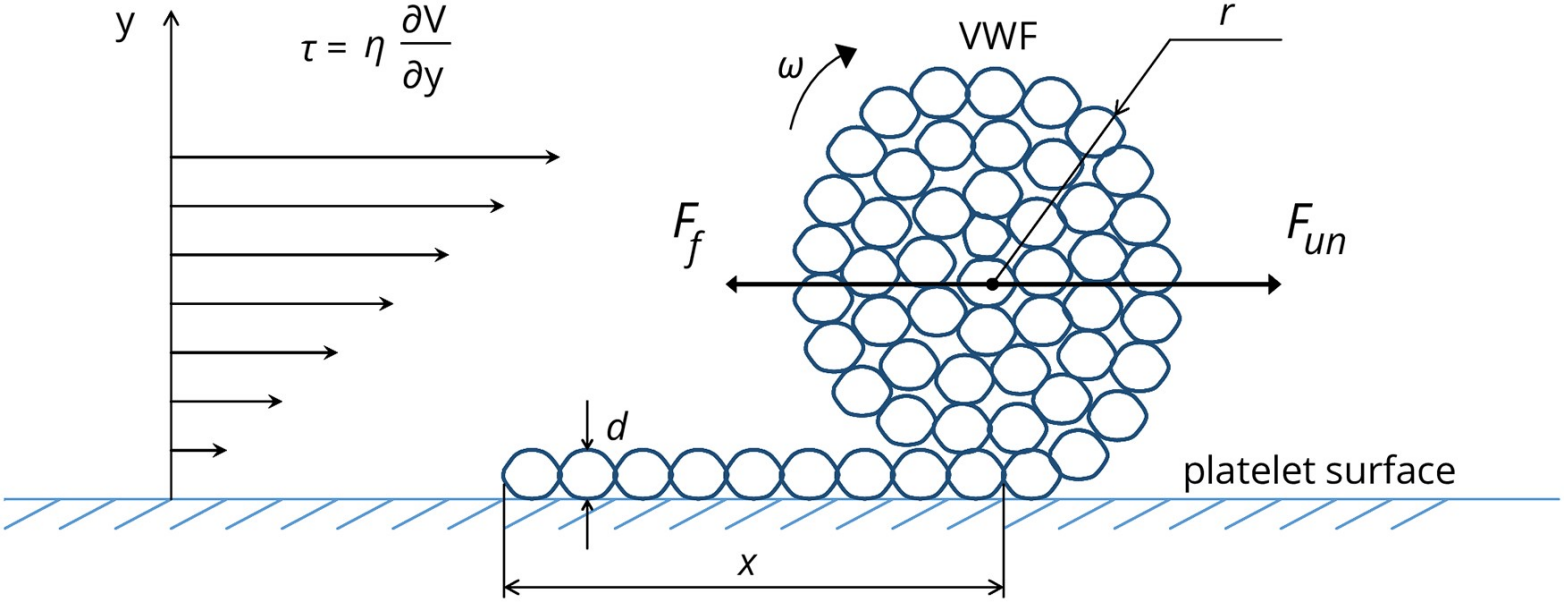
Partially unfolded VWF molecule in shear flow. *r* denotes the radius of the VWF globular part, *x* corresponds to the length of the unfolded part on the platelet surface (“tail”) of the molecule, *d* denotes the characteristic size of the VWF monomeric subunit, *F*_*f*_ denotes the force due to effective surface tension that tends to fold a VWF molecule, *F*_*un*_ is the force that unfolds a VWF molecule under the action of shear stress *τ*, *η* denotes blood viscosity, and *ω* reflects the rotational speed of the globular part of the molecule. The symbol dV/dy denotes a velocity gradient perpendicular to the direction of the flow (i.e. shear rate). Adapted from [35].

Consider a VWF molecule grafted at a definite point on a platelet surface. Assume that it consists of *N* subunits of size *d* each, where the VWF contour length is *L* ≡ *N* ⋅ *d*. The molecule can be unfolded under shear stress acting on it in flow. Let the radius of the globular part of the molecule be denoted by *r*, while *x* denotes a length of the unfolded part of the molecule (“tail”, Fig 2). The basic equation describing the VWF molecule dynamics on the platelet surface under unsteady shear stress may be written in accordance with the rules of analytical mechanics:

$$\frac{d}{dt}\left(J_{r}\omega \right)=M_{un}-M_{f}$$ (2)

where symbol *J*_*r*_ denotes the moment of inertia of the globular part of the VWF molecule and *ω* displays rotational speed of globular part of the molecule. *M*_*un*_ ≡ *F*_*un*_*r* is the moment of unfolding force *F*_*un*_ due to the action of shear stress on VWF molecules from the flow side, tending to unfold the molecule, and *M*_*f*_ ≡ *F*_*f*_*r* is the moment of folding force *F*_*f*_, tending to return the molecule to a globular conformation.

Using a simple approximation [36,37], the unfolding force *F*_*un*_ can be evaluated as:

$$F_{un}=k\pi r^{2}\tau$$ (3)

where *τ* = *τ*(*t*) denotes the shear stress value and *k* represents a dimensionless proportionality coefficient. Folding force is caused by the presence of effective “surface tension” that tends to return a molecule to its most compact shape (globular state). The absolute value of the folding force is defined as follows (S1 Text):

$$F_{f}=\sigma \pi d\left(1-\frac{d}{2r}\right)$$ (4)

where *σ* corresponds to the coefficient of the effective “surface tension”.

Explicit expressions for the moment of inertia *J*_*r*_ and angular speed of the globular part of the molecule *ω* can be rewritten in the following forms (for details, S1 Text):

$$J_{r}=\frac{112r^{5}}{15d^{3}}m_{0}$$ (5)

$$\omega =\frac{1}{r}\frac{dx}{dt}=-\frac{16r}{d^{2}}r_{t}^{\prime }$$ (6)

The symbol *m*_0_ in Eq (5) corresponds to the mass of a single monomeric subunit of the VWF molecule. Taking into account Eqs (3)–(6), the equation of motion (2), describing the conformational dynamics of the VWF macromolecule, can be easily transformed to the following form (S1 Text):

$$\frac{4m_{0}}{15\sigma \pi }{\left[{\left(\frac{2r}{d}\right)}^{7}\right]}_{tt}^{\prime \prime }=-\frac{k\tau d}{4\sigma }{\left(\frac{2r}{d}\right)}^{3}+\left(\frac{2r}{d}\right)-1$$ (7)

where double prime denotes the second derivative of the expression with respect to time. Using the designation *q* = (2*r*/*d*)^7^, the equation for VWF folding/unfolding (7) is converted to the following dimensionless form:

$$q_{\tilde{t}\tilde{t}}^{\prime \prime }=-\tilde{\tau }q^{3/7}+q^{1/7}-1$$ (8)

where $\tilde{t}\equiv t\sqrt{15\sigma \pi /4m_{0}}$ represents a dimensionless value, corresponding to time, while $\tilde{\tau }\equiv k\tau d/4\sigma$ denotes the dimensionless shear stress. Note that the dynamic dimensionless variable $q\equiv q(\tilde{t})$ can vary from *q* = *q*_*m*_ = (3*N*/2)^7/3^ (folded state) to *q* = 0 (totally unfolded state). One could receive the following relation between the order parameter *u* = *x*/*L* and the variable *q*: *u* = 1 − (*q*/*q*_*m*_)^3/7^. Obviously, *u* = 0 if the VWF molecule is in its folded state, and *u* = 1 if VWF is in a totally unfolded state.

Eq (8) can be formally considered as a motion equation of a material point having a unit mass and coordinate *q* in a potential force field:

$${\tilde{U}}_{\tilde{\tau }}\left(q\right)=\frac{7}{10}\tilde{\tau }q^{10/7}-\frac{7}{8}q^{8/7}+q$$ (9)

Note that the first term on the right-hand side of Eq (8) describes an action of shear stress $\tilde{\tau }$ and in principle can be time dependent (for instance, during platelet motion in vessels with a variable cross-section). The conformational states of molecules adsorbed on platelet surfaces will change in accordance with Eq (8) during platelet motion from low to high shear stress zones and back.

## Results

### Analysis of the phase portrait and formulation of the critical condition of shear-induced platelet activation

The central question is as follows: what are the conditions for irreversible conformational unfolding of VWF? Looking for the answer to this question, it seems appropriate to build phase portraits of the considered system in accordance with standard methods of analytical mechanics [38].

**Fig 3:**
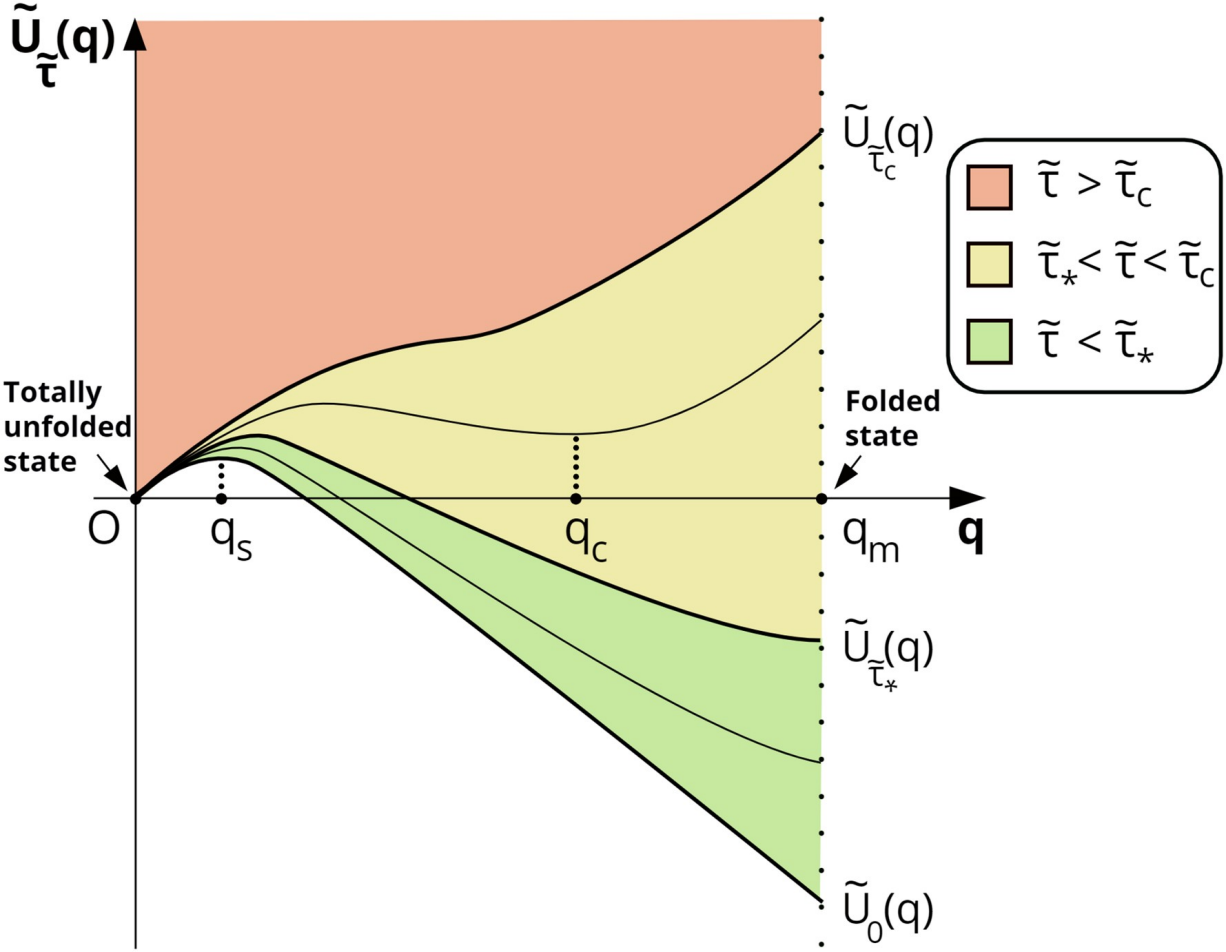
Potential energy profile ${\tilde{U}}_{\tilde{\tau }}(q)$ at various $\tilde{\tau }$ values. Three characteristic regions are shown. Any potential curve at $\tilde{\tau }>{\tilde{\tau }}_{c}$, ${\tilde{\tau }}_{*}<\tilde{\tau }<{\tilde{\tau }}_{c}$ and $\tilde{\tau }<{\tilde{\tau }}_{*}$ lies in the domain denoted in red, yellow and green, respectively. Points *q* = *q*_*s*_ and *q* = *q*_c_ are points of maximum and minimum potential energy ${\tilde{U}}_{\tilde{\tau }}(q)$, respectively. Bold lines correspond to potential energy curves for $\tilde{\tau }=0$, $\tilde{\tau }={\tilde{\tau }}_{*}$, and $\tilde{\tau }={\tilde{\tau }}_{с}$ and are define as ${\tilde{U}}_{0}(q)$, ${\tilde{U}}_{{\tilde{\tau }}_{*}}(q)$ and ${\tilde{U}}_{{\tilde{\tau }}_{с}}(q)$, respectively.

Profiles ${\tilde{U}}_{\tilde{\tau }}(q)$ corresponding to the potential energy drawn in accordance with Eq (9) are shown in Fig 3. The potential energy curves for the three sets of $\tilde{\tau }$ values have different numbers of extrema.

When the value of shear stress satisfies the condition ${\tilde{\tau }>\tilde{\tau }}_{c}$, all the corresponding curves of potential energy have only one minimum at *q* = 0 (Fig 3, red region). This means that in quite intense steady shear flows, VWF molecules should be in totally unfolded states. The second possibility is realized when ${\tilde{\tau }}_{*}<{\tilde{\tau }<\tilde{\tau }}_{c}$ (yellow region). In this region, all curves of potential energy have two local minima: one at *q* = 0 and the other at *q* = *q*_*c*_ belonging to the region (*q*_*s*_, *q*_*m*_). This means that VWF molecules should be either in the completely unfolded state (*q* = 0) or in a partially unfolded state (*q* = *q*_*c*_). Finally, when $\tilde{\tau }<{\tilde{\tau }}_{*}$ (green region), the curves of potential energy have two local minima: one at *q* = 0 and the other at *q* = *q*_*m*_. In relevant flows, VWF molecules should be either in the completely unfolded state (*q* = 0) or in the folded state (*q* = *q*_*m*_). It should be noted that under the condition ${\tilde{\tau }<\tilde{\tau }}_{c}$, every curve of potential energy also has one local maximum at *q* = *q*_*s*_.

It is clear that potential energy minima correspond to the states in which the dynamic system has a tendency to remain if left to itself. A local maximum of potential energy defines the height of a barrier that the system needs to overcome for transition from one local stable state (*q* = *q*_*m*_) to another (*q* = 0).

**Fig 4:**
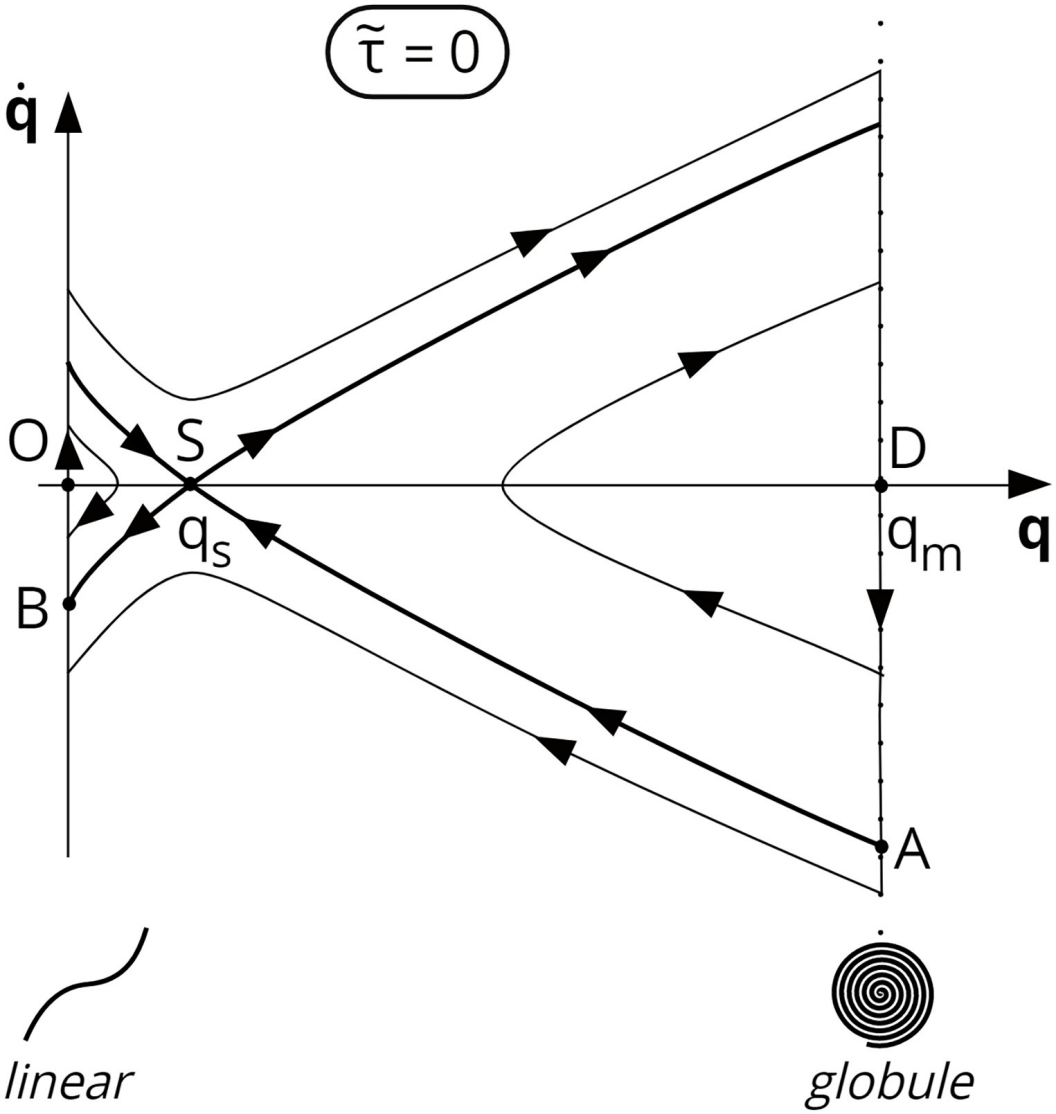
Schematic view of the phase portrait at $\tilde{\tau }=0$. The value *q* = 0 corresponds to the unfolded state of the VWF molecule; and *q* = *q*_*m*_, to the folded state. *q* = *q*_*s*_ is a saddle point, and the curve ASB denotes the separatrix.

The phase portrait of the system for the case $\tilde{\tau }=0$ is shown in Fig 4 (topological transformations of phase portraits for abovementioned intervals of shear stress $\tilde{\tau }>0$ are shown in S2-1 Fig, S2 Text). It is clear that to change the molecular conformation from the initially folded state *q* = *q*_*m*_, $\dot{q}=0$ (point D) to the totally unfolded state *q* = 0, the momentum imparted to the molecule must be rather strong. The corresponding image point, representing the system state, should fall at a definite moment below the separatrix AS (shown in Fig 4 by a bold line).

**Fig 5:**
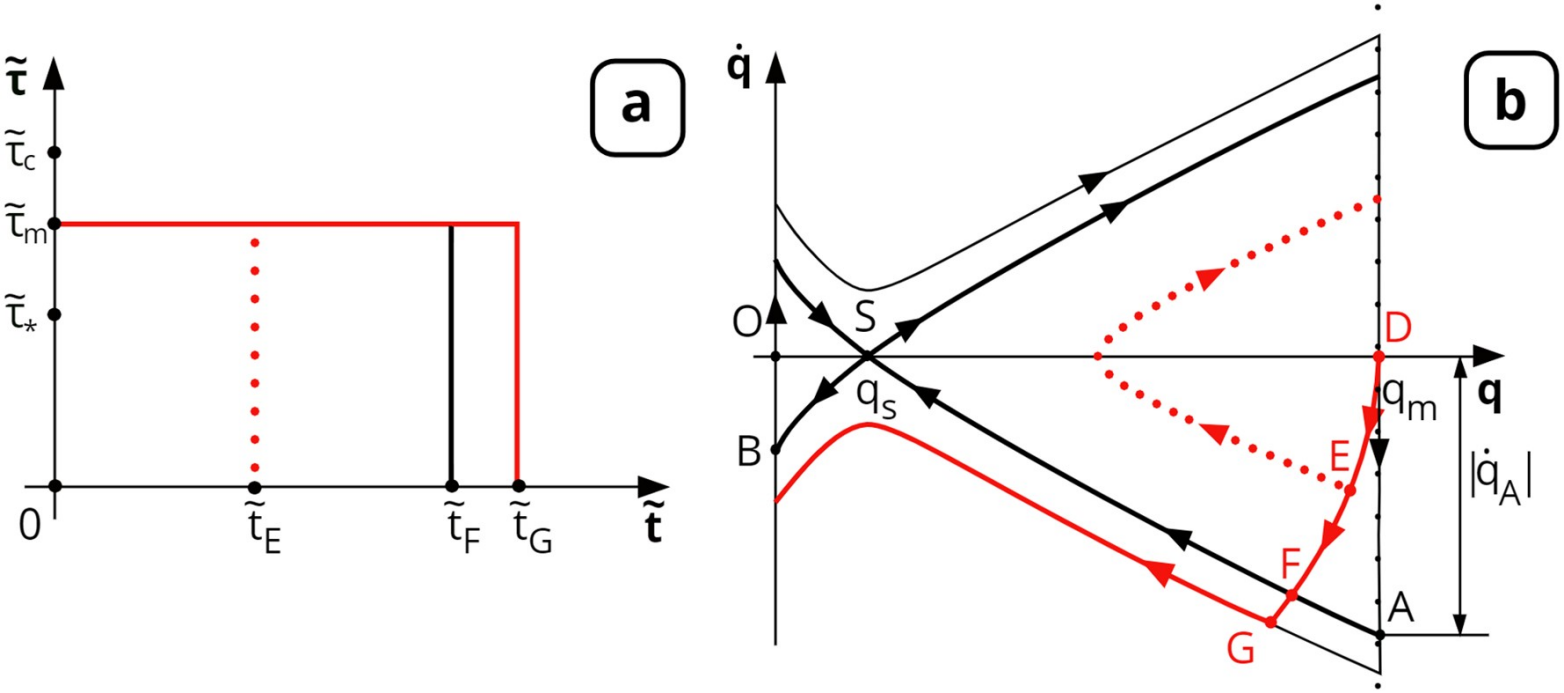
VWF molecule unfolding under the action of a rectangular shear stress profile with various durations. (a) Shear stress impulse $\tilde{\tau }(\tilde{t})$ is shown in red, ${\tilde{\tau }}_{m}$ is its amplitude, and ${\tilde{t}}_{G}$ is its duration. (b) Phase trajectory of the solution (Eq (8)) is illustrated by the red line for the shear stress profile from Fig 5a; initial conditions: *q* = *q*_*m*_, $\dot{q}=0$. ${\tilde{t}}_{E}$, ${\tilde{t}}_{F}$ and ${\tilde{t}}_{G}$ are time moments when the image point reaches points E, F and G, respectively. If the duration of the shear stress impulse ${\tilde{t}}_{G}$ is greater than that of ${\tilde{t}}_{F}$, the trajectory crosses the part of the separatrix AS, and the molecule can unwind to its full length. $\left|{\dot{q}}_{A}\right|$ is the absolute value of the momentum at point A.

One of the possible ways to impart the momentum $\dot{q}$ to the molecule is to expose it to shear stress. Let us consider the response of the VWF molecule to a rectangular shear stress impulse (Fig 5). After application of a rectangular shear stress impulse of amplitude ${\tilde{\tau }}_{m}$ and duration ${\tilde{t}}_{G}$ (Fig 5a), the phase trajectory starting from point D crosses the separatrix AS (Fig 5b) at a certain moment ${\tilde{t}}_{F}$. All integral curves passing below the separatrix AS (Fig 5b) reach the left boundary of the physically acceptable region (*q* = 0) below point B. That is, all these curves lead to the completely unfolded state of VWF molecules on the platelet surface. Clearly, VWF molecules that go to the unfolded state (*q* = 0) will have nonzero momentum $|\dot{q}|>|{\dot{q}}_{B}|\neq 0$ at the time of their complete unfolding to *q* = 0. Moreover, the lower the situated corresponding integral curve at the phase portrait, the larger the absolute value of the “residual” momentum.

Further evolution of the unfolded VWF molecule depends on the boundary conditions at *q* = 0. In principle, there are three possibilities depending on the particularities of platelet surface interaction with VWF molecules (all possibilities mentioned above are discussed in detail in S2 Text): absolutely inelastic, partially elastic and absolutely elastic collision. The absolutely elastic boundary condition corresponds to the situation when there is no energy loss in a collision. VWF multimer being totally unfolded retains an opportunity to return back to its initial folded state in this case. The absolutely inelastic boundary condition corresponds to irreversible adhesion of VWF molecules on the platelet surface after unfolding. We suppose that some intermediate situation takes place in a real situation corresponding to a partially elastic boundary (see S2 Text for details).

**Fig 6:**
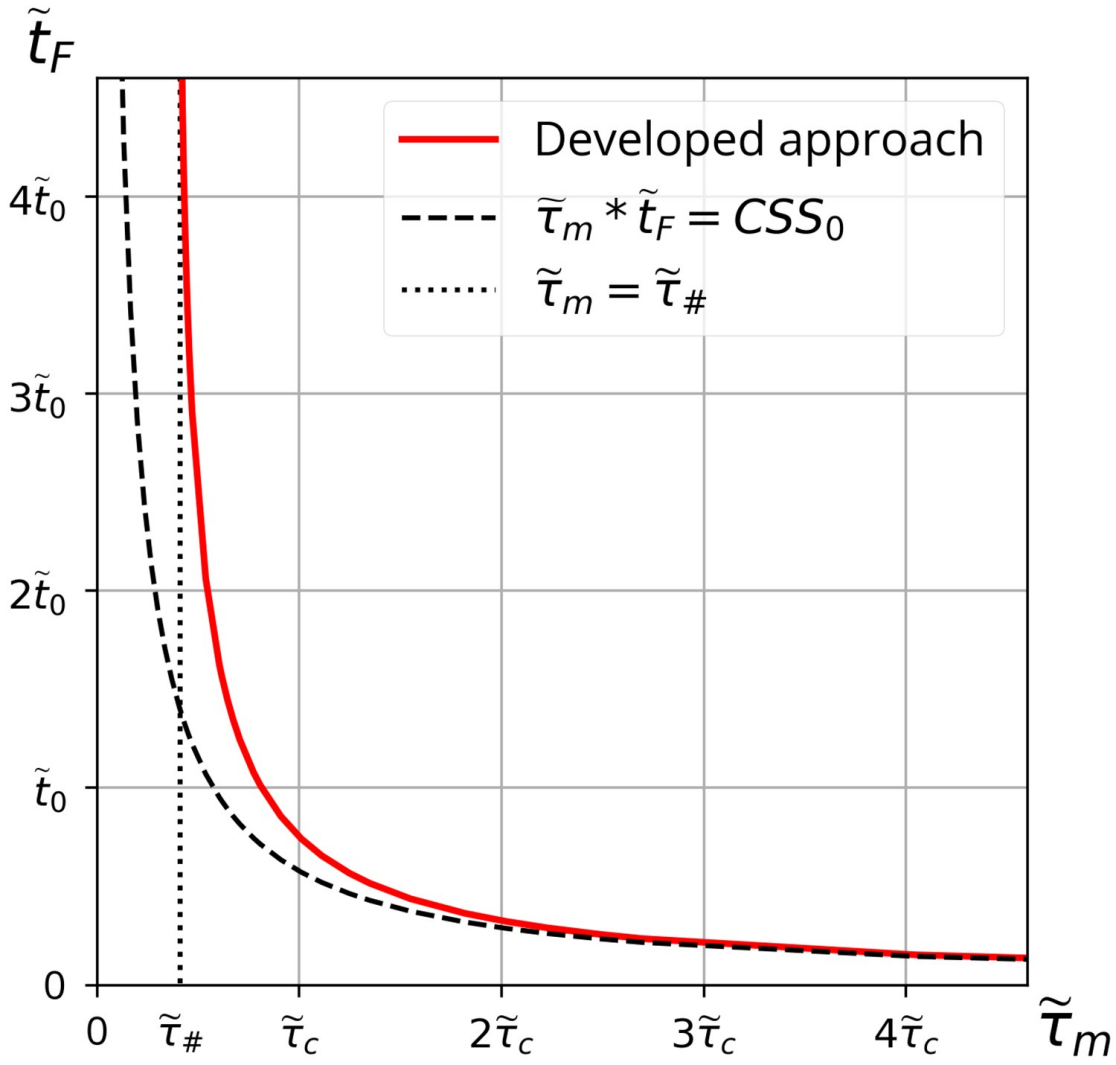
Dependence of critical time duration ${\tilde{t}}_{F}$ on amplitude ${\tilde{\tau }}_{m}$ of a rectangular shear stress impulse. The solid curve corresponds to the dependence ${\tilde{t}}_{F}({\tilde{\tau }}_{m})$ obtained in the framework of developed approach (S4 Text for details). The dotted line ${\tilde{\tau }}_{m}={\tilde{\tau }}_{\#}$ is a vertical asymptote of the curve ${\tilde{t}}_{F}({\tilde{\tau }}_{m})$. The curve ${\tilde{t}}_{F}({\tilde{\tau }}_{m})$ is asymptotically tending to the dashed curve with increasing ${\tilde{\tau }}_{m}$. The values of ${\tilde{t}}_{F}$ are measured in units of characteristic unfolding time ${\tilde{t}}_{0}$ (S4 Text). The value of *CSS*_0_ is given by Eq (16). All curves were built for the case of *N* = 36.

Numerical investigation of the critical cumulative shear stress condition achieved for the case of a rectangular shear impulse is discussed in detail in S4 Text. In particular, the dependence of ${\tilde{t}}_{F}$ on the value of applied shear stress ${\tilde{\tau }}_{m}$ (for any definite value of *N*) was found (Fig 6).

The dependence ${\tilde{t}}_{F}({\tilde{\tau }}_{m})$ is shown in Fig 6. It has a vertical asymptote ${\tilde{\tau }}_{m}={\tilde{\tau }}_{\#}$. At the same time, it is clear that within a fairly wide range of amplitudes ${\tilde{\tau }}_{m}({\tilde{\tau }}_{m}>2{\tilde{\tau }}_{c})$, the curve may be approximated by the expression ${\tilde{\tau }}_{m}{\tilde{t}}_{F}=CSS_{0}$. The value of *CSS*_0_ is determined by Eq (16). The dependence of ${\tilde{\tau }}_{\#}$ as a function of *N* is given in S4-3 Fig (S4 Text).

Indeed, it is easily seen from Fig 6 that the solid line (reflecting rigorous condition of VWF unfolding) lies above the dashed line relevant to the curve ${\tilde{\tau }}_{m}{\tilde{t}}_{F}=CSS_{0}$. Thus, condition ${\tilde{\tau }}_{m}{\tilde{t}}_{F}>CSS_{0}$ should be treated as the necessary condition, while the rigorous sufficient condition is determined by the numerical solution to Eq (8) represented by the solid line in Fig 6. The two lines practically coincide with each other when ${\tilde{\tau }}_{m}>2{\tilde{\tau }}_{c}$. However, in the interval ${\tilde{\tau }}_{m}\in ({\tilde{\tau }}_{\#},{2\tilde{\tau }}_{c})$, the discrepancy between the lines is significant. Moreover, it can be seen from Fig 6 that total unfolding of VWF induced by a shear stress less than the threshold value ${\tilde{\tau }}_{m}<{\tilde{\tau }}_{\#}$ is not possible at any finite time interval. Thus, relevant platelet activation should not take place.

### Derivation of the explicit expression for the *CSS*_0_ as a function of VWF multimer size

It is worth to notice that the approach developed in the current work makes it possible to investigate VWF unfolding and relevant platelet activation not only for a rectangular profile of shear stress but also for an arbitrary form of shear stress *τ*(*t*) as a function of time. Indeed, below, we demonstrate analytically how the critical condition for cumulative shear stress can be derived.

Consider a VWF molecule passing through a high shear stress zone. ${\dot{q}}_{in}$ and ${\dot{q}}_{out}$ correspond to momentum at moments ${\tilde{t}}_{in}$ and ${\tilde{t}}_{out}$, respectively, when platelets go in and out of the high shear stress zone. Suppose that the VWF molecule is in a folded state at the initial moment ${\tilde{t}}_{in}$. That formally means that $q({\tilde{t}}_{in})=q_{m}$ and ${\dot{q}}_{in}\equiv \dot{q}({\tilde{t}}_{in})=0$. Looking at the phase portrait (Fig 5b), it becomes clear that the sufficient condition of total VWF unfolding has the form:

$$\left|{\dot{q}}_{out}\right|>\left|{\dot{q}}_{A}\right|$$ (10)

It is easy to obtain the value of the momentum at point A (S3 Text):

$$\left|{\dot{q}}_{A}\right|=\sqrt{2\left({\tilde{U}}_{0}\left(q_{s}\right)-{\tilde{U}}_{0}\left(q_{m}\right)\right)}$$ (11)

The momentum value ${\dot{q}}_{out}$, that a VWF molecule obtained when passing through the high shear stress zone, can be found by integrating Eq (8) with respect to time:

$${\dot{q}}_{out}-{\dot{q}}_{in}=\int_{{\tilde{t}}_{in}}^{{\tilde{t}}_{out}}\ddot{q}d\tilde{t}=\int_{{\tilde{t}}_{in}}^{{\tilde{t}}_{out}}\left(-\tilde{\tau }q^{\frac{3}{7}}+q^{\frac{1}{7}}-1\right)d\tilde{t}$$ (12)

Taking into account that ${\dot{q}}_{in}=0$, Eq (12) can be transformed into the following form:

$$\left|{\dot{q}}_{out}\right|=\int_{{\tilde{t}}_{in}}^{{\tilde{t}}_{out}}q^{\frac{3}{7}}\tilde{\tau }\left(\tilde{t}\right)d\tilde{t}-\int_{{\tilde{t}}_{in}}^{{\tilde{t}}_{out}}\left(q^{\frac{1}{7}}-1\right)d\tilde{t}$$ (13)

By substituting Eqs (11) and (13) into inequality (10) and keeping in mind that *q* > *q*_*s*_ = 1 under $\tilde{\tau }=0$ the necessary condition for complete VWF unfolding takes the form (for details S3 Text):

$$\int_{{\tilde{t}}_{in}}^{{\tilde{t}}_{out}}q^{\frac{3}{7}}\tilde{\tau }\left(\tilde{t}\right)d\tilde{t}>\sqrt{2\left({\tilde{U}}_{0}\left(q_{s}\right)-{\tilde{U}}_{0}\left(q_{m}\right)\right)}$$ (14)

Taking into account that the values of the dynamical variable *q*(*t*) during VWF unfolding are always less than its maximal value *q*_*m*_ (*q*(*t*) < *q*_*m*_) the following inequality can be obtained:

$$\int_{{\tilde{t}}_{in}}^{{\tilde{t}}_{out}}\tilde{\tau }\left(\tilde{t}\right)d\tilde{t}>\frac{1}{q_{m}^{\frac{3}{7}}}\sqrt{2\left[{\tilde{U}}_{0}\left(q_{s}\right)-{\tilde{U}}_{0}\left(q_{m}\right)\right]}\equiv CSS_{0}$$ (15)

In other words, to unfold a VWF multimer, the value of the cumulative shear stress must be at least larger than a definite critical value *CSS*_0_ standing on the right-hand side of inequality (15). Expression (15) represents the necessary but not sufficient condition of VWF unfolding on the surface of platelets.

The developed approach gives us the opportunity not only to find an expression for the condition of the cumulative shear stress (Eq (15)) but also to find the dependence of *CSS*_0_ as a function of *N*. Indeed, taking into consideration that *q*_*s*_ = 1, *q*_*m*_ = (3*N*/2)^7/3^ and that the expression for potential energy has a form ${\tilde{U}}_{0}(q)=-(7{/8)q}^{8/7}+q$, the value of the critical cumulative shear stress is given by the following formula:

$$CSS_{0}={\left(\frac{3}{2}N\right)}^{\frac{1}{3}}\sqrt{\frac{7}{4}+\frac{1}{4}{\left(\frac{3}{2}N\right)}^{-\frac{8}{3}}-2{\left(\frac{3}{2}N\right)}^{-\frac{1}{3}}}$$ (16)

Eq (16) represents an explicit expression for the dependence of the critical cumulative shear stress *CSS*_0_ value on the multimeric size of VWF molecules. Thus, the value of the critical cumulative shear stress is an increasing function of the VWF multimer size *N*.

## Discussion

In the current work, we propose an approach for the analytical derivation of the platelet activation conditions under unsteady shear stress (expressions (15) and (16)). The approach is based on the idea that the conformation of VWF molecules which are grafted on platelet surfaces can be dynamically changed under unsteady flow. VWF unfolding results in the increase of an efficient connection between VWF monomer units and GPIb receptors on the platelet surface. When the cumulative shear stress exceeds a critical value (inequality (15)), the platelet is primed for activation. As a result of the proposed approach, the dependence of the threshold value of cumulative shear stress on VWF multimer size was derived (Eq (16)).

The problem of shear-induced unfolding of VWF molecules and its presumable effect on platelet activation has been intensively studied during the last four decades [21,39–41]. Most of the previous studies focused on the investigation of VWF-platelet interactions under steady shear stress. Particularly, the platelet activation was analysed in microcirculation [18,42]. In contrast, in stenotic regions of large vessels platelets are exposed to unsteady shear stress, rapidly varying along platelet trajectories. For this reason, in the present article we focused on the investigation of unsteady shear-induced platelet activation (SIPAct) phenomena in large vessels.

Shear gradients, as well as the duration of exposure to high shear stress, can influence the onset of SIPAct in the presence of VWF molecules [18,28,31,34,43,44]. The haemostatic activity of VWF directly correlates with its size [39,45]. To the best of our knowledge, no mathematical expression describing SIPAct relation to the characteristics of dynamic shear stress (amplitude and duration) and the length of VWF molecules has been suggested. Corresponding formulas are derived in this work (Eqs (15) and (16)).

The approach allowed us to derive the equation for VWF folding/unfolding on a platelet surface under dynamic shear stress. It was shown that conformational changes of VWF during platelet movement through a high shear stress zone can be calculated by numerical integration of Eq (8). This equation may be used for the estimation of shear-induced platelet activation under unsteady flow conditions. Particularly, basing on Eq (8), condition (15) for complete VWF unfolding on the platelet surface under dynamic shear was obtained. According to this condition, any value of cumulative shear stress less than the threshold value should not lead to full unfolding of VWF.

The analytical expression for the critical cumulative shear stress as a function of VWF multimer size (Eq (16)) was derived. When passing through a high shear zone, VWF experiences an “impact” that may lead to full unfolding. Eq (16) shows that large multimers need stronger “impact” for full unfolding than smaller multimers.

The unfolding of VWF under the action of a rectangular shear stress profile was investigated numerically (Fig 5). The obtained results indicate that in the case of short but rather strong shear impulses (${\tilde{\tau }}_{m}>2{\tilde{\tau }}_{c}$), condition (15) may be used as a high accuracy approximation for the condition of full unfolding of VWF on the platelet surface, resulting in subsequent priming of the platelet (Fig 6).

Condition (15) is suitable for describing shear-induced platelet activation in laminar blood flow. In such a flow, it is always possible to distinguish trajectories of individual platelets and to track how shear stress is changing along trajectories. The finding of dependencies of shear stress along a trajectory in laminar flow seems to be a standard computational fluid dynamics (CFD) task [46]. In particular, this task can be routinely solved for blood vessels with variable cross-sections [24,25]. Once the dependency is obtained, one can easily check the fulfilment of condition (15) for any selected platelet trajectory.

The applicability of the developed approach to the investigation of shear-induced platelet activation in turbulent blood flow remains unclear and requires further study.

In further analysis of laminar blood flow, it is worth taking into account that hydrodynamic conditions corresponding to shear-induced platelet activation may be divided into two groups [47–50]. The first group includes situations where shear stress is steady (for instance, as in a cone-and-plate viscometer) and exposure time lies within the range from several seconds to minutes [39,40,47]. The second group includes regimes in locally stenotic blood vessels *in vivo* and constricted artificial channels *in vitro*. The current experimental data show that the characteristic time during which platelets are exposed to high shear stress varies from a few milliseconds to fractions of seconds [23,51], while the characteristic amplitude of shear stress may exceed 1000 (*dyn*/*cm*^2^) [52,53].

According to the approach developed in this paper, the difference between the two groups is related to the value of the shear stress gradient, i.e., the derivative of $\tilde{\tau }=\tilde{\tau }(\tilde{t})$. In cases where the shear stress gradient is sufficiently small, the shear stress along the platelet trajectory will vary more slowly than the conformation of the VWF molecule. Thus, the value of shear stress could be considered a parameter in Eq (8). Platelet activation under such conditions takes place in a parametric regime. In cases where the shear stress gradient is sufficiently large, VWF conformational changes will delay after shear stress variations. In other words, rapid platelet movements through the stenotic region result in an impulsive hydrodynamic impact on VWF. Under impulsive impacts, the shear stress in Eq (8) could be treated as a dynamical variable. Therefore, SIPAct under such conditions occurs in a dynamic regime.

The corresponding momentum acquired by the VWF may be subcritical (less than ${|\dot{q}}_{A}|$) or overcritical (more than ${|\dot{q}}_{A}|$)). In the second case, such momentum is enough for full unfolding of VWF on the platelet surface (Fig 5). The relevant condition (10) formally may be considered a sufficient condition for the total unfolding of VWF, which seems to be a necessary condition for the priming of platelets.

The developed approach allows us to analyse both the impulsive and quasistatic evolution of the VWF conformation on the platelet surface. In the case of impulsive evolution, one could say that the hydrodynamic environment around platelets changes faster than the conformation of VWF molecules on their surface. On the other hand, when quasistatic evolution takes place, VWF molecules can adapt their conformations under external actions. The degree of their unfolding follows the changes in shear stress. The conformational state of VWF may be established from the equilibrium conditions derived from Eq (8).

In cases where shear stress impacts act during a very short period of time, VWF should relax to new equilibrium states over some period of time. In the current work, our purpose was limited only to an estimation of phase portrait transformations corresponding to the dynamics of the VWF molecule to equilibrium states under small dissipative effects (S2 Text).

It is well known that shear stress reaches the highest values in the near-wall region of vessels [54] The concept of wall shear stress (WSS) has become widespread not only among CFD specialists but also among physicians [55,56]. To interpret the results obtained in this paper, it should be noted that the velocity of platelets moving near the vessel wall tends to zero under no-slip conditions [57]. For all trajectories lying adjacent to the vessel wall, dependencies of shear stress are slowly varying functions of time. This means that the conditions for VWF unfolding in near-wall regions can be considered parametric.

The farther from the wall the trajectory of motion is, the shorter the time it takes for a platelet to cross through a locally stenotic region. Thus, short-term impulsive action from the side of the flow on VWF molecules should take place. The question is whether this kind of impulsive action may lead to full unfolding of VWF on platelet surfaces resulting in subsequent priming of the platelets. The answer is given by condition (15) obtained in this paper. Consequently, within the framework of our approach, both parametric and dynamic regimes of SIPAct can simultaneously be realized in the same stenotic artery but at a different distance from the vessel wall.

It is worth discussing the extent to which the obtained results may be relevant to problems of thrombosis initiation in different blood-wetted devices. For the moment, we can formulate a few conclusions concerning cumulative shear stress. At high shear stress (${\tilde{\tau }}_{m}>2{\tilde{\tau }}_{c}$), approximation condition (1) is quite suitable for the estimation of device thrombogenicity. When the shear stress lies in the range of ${\tilde{\tau }}_{\#}<{\tilde{\tau }}_{m}<2{\tilde{\tau }}_{c}$, condition (1) could be treated as necessary but insufficient for SIPAct. If the shear stress is less than the threshold level ${\tilde{\tau }}_{m}<{\tilde{\tau }}_{\#}$, condition (1) is unusable for the evaluation of platelet activation.

The existence of a lower shear stress threshold above which SIPAct may take place is intensively discussed in some articles dedicated to the problem of thrombogenicity in blood-wetted devices [58,59] and in stenotic vessels [25,31]. The obtained results give the answer as to how the value of the lower threshold should depend on the multimeric size *N* of VWF molecules (S4-3 Fig of S4 Text).

Several experiments were conducted to study platelet function under high shear stress in different microfluidic platforms [17,18,22,43,60,61]. Particularly, the influence of dynamic shear stress on platelet activation was investigated [28,34,42]. We think that a critical value of cumulative shear stress as a function of multimer size could be measured in the framework of the microfluidic experiments. The regulation of the size of VWF molecules in relevant experimental conditions may be fulfilled with the aid of ADAMTS-13 [4,62,63]. Appropriate experiments in large-scale vessels also could be done. It will give an opportunity for the direct confirmation/falsification of our theoretical results.

It is worth noting that in general, the developed approach is phenomenological. It describes the SIPAct phenomena under unsteady shear stress rather roughly, in contrast to the detailed molecular dynamics approach [64,65]. Nevertheless, the developed approach opens the possibility for estimating the relative impact of parametric and dynamic regimes of VWF unfolding on shear-induced platelet activation in a variety of arterial and venous vessels of the human blood circulatory system. Indeed, an explicit formula for the critical cumulative shear stress as a function of VWF multimer size is found (Eq (16)). This expression is important for the regulation of shear-induced platelet activation not only by biomechanical factors (WSS, arterial pressure, distensibility of vessel walls, etc.) [19,66], but also by several biochemical and pharmacological agents affecting VWF multimer size (ADAMTS-13, N-acetylcysteine) [4,67–69].

We believe that these findings may be used to improve clinical protocols for haemostasis correction.

## Supporting information

S1 Text Derivation of the dynamic VWF unfolding equation. (PDF)

S2 Text Analysis of solution behaviour at physical boundaries. (PDF)

S3 Text Derivation of the critical condition of the total VWF unfolding. (PDF)

S4 Text VWF unwinding under the action of rectangular shear stress profile. (PDF)
