# Supplementary material for: Platelet activation via dynamic conformational changes of von Willebrand factor under shear
Source: PLoS One. 2020 Jun 11;15(6):e0234501. doi: 10.1371/journal.pone.0234501 (PMC7289367; doi:10.1371/journal.pone.0234501)
Supplement: S1 Text — (PDF) [file pone.0234501.s001.pdf]

S1 Text. Derivation of the dynamic VWF unfolding equation.

Consider a VWF molecule that is grafted onto a platelet surface. The molecule is in a globular state under low shear conditions. If the shear stress exceeds the definite threshold value, the molecule begins to unwind [S1.1,S1.2]. Let the length of the unfolded part of the molecule ("tail") be denoted by x and the radius of the globular part by r (S1-1 Fig). Due to conservation of VWF volume during unwinding, the following relationship between x and r is always valid:

$$L\frac{\pi d^2}{4} = x\frac{\pi d^2}{4} + \frac{4}{3}\pi r^3 \qquad \text{(S1-1)}$$

where $L \equiv Nd$ is the contour length of VWF, N is the total number of monomeric subunits and d is the size of one subunit. It follows from equation (S1-1) that $x = L$ and $r = 0$ in the completely unfolded state and $x = 0$ and $r = R$ in the folded state, where $R = (d/2)\sqrt[3]{3N/2}$ is the maximum radius of a VWF globule.

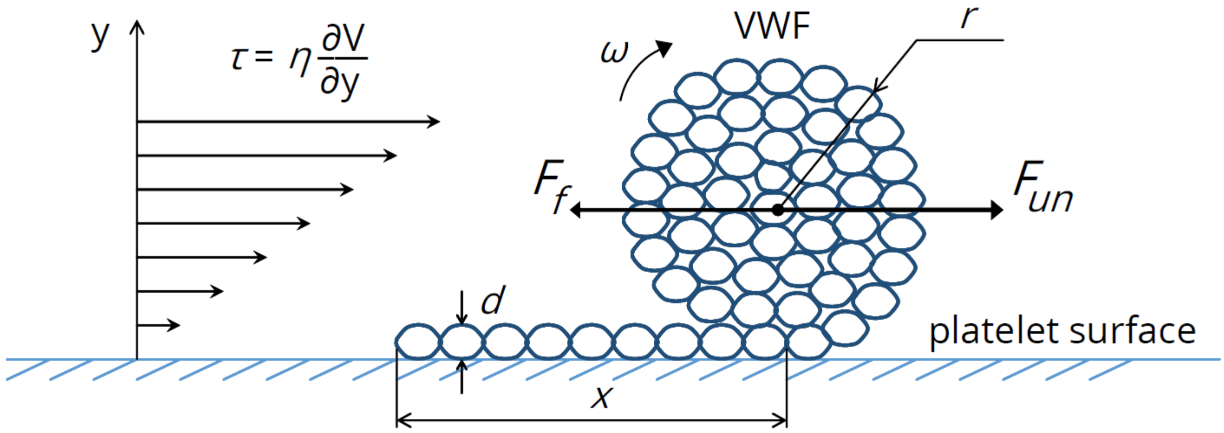

S1-1 Fig. Partially unfolded VWF molecule in shear flow. r denotes the radius of the VWF globular part, x corresponds to the length of the unfolded part on the platelet surface ("tail") of the molecule, d denotes the characteristic size of the VWF monomeric subunit, F_f denotes the force due to effective surface tension that tends to fold a VWF molecule, F_{un} is the force that unfolds a VWF molecule under the action of shear stress τ, η denotes blood viscosity, and ω reflects the rotational speed of the globular part of the molecule. Adapted from [S1.3].

Dividing the left- and right-hand sides of equation (S1-1) by the characteristic volume of one monomeric subunit $V_0 = \pi(d/2)^3$, the expression takes the following form:

$$\frac{L}{d} = \frac{x}{d} + \frac{16}{3}\frac{r^3}{d^3} \tag{S1-2}$$

Here, $n_x \equiv x/d$ and $n_r \equiv 16r^3/3d^3$ are the numbers of monomeric subunits in the "tail" and globular parts, respectively.

In the current work, we assumed that only two forces act on a grafted VWF molecule. The folding force F_f is caused by the surface tension σ [S1.4]. The surface energy E of VWF is proportional to the surface area of the molecule:

$$E = \sigma(\pi x d + 4\pi r^2) \tag{S1-3}$$

The first derivative of E on x gives the expression for the absolute value of F_f:

$$F_f = \sigma\pi d(1 - \frac{d}{2r}) \tag{S1-4}$$

The unfolding force F_{un} is proportional to the shear stress τ and molecular cross-section area [S1.5]:

$$F_{un} = k\pi r^2\tau \tag{S1-5}$$

where k is a dimensionless proportionality coefficient.

The dynamics of VWF molecule folding/unfolding in the blood flow are described by the equation:

$$(J_r\omega)'_t = (F_{un} - F_f)r \tag{S1-6}$$

where J_r is the molecular moment of inertia of the globular part and ω is its rotational speed. The prime symbol means the first derivative of the expression in time. The globular part consists of n_r monomeric subunits with a mass m_0 (equation (S1-2)). The expression for J_r with respect to the instant centre of rotation can be written as follows:

$$J_r = \frac{7}{5}(m_0 n_r)r^2 \tag{S1-7}$$

In turn, the value of rotational speed ω is given by the formula:

$$\omega = \frac{1}{r} x_t' \tag{S1-8}$$

Keeping in mind that $n_r \equiv 16r^3/3d^3$ and using equation (S1-2), the expressions for J_r and ω take the following forms:

$$J_r = \frac{112}{15}\frac{m_0}{d^3} r^5 \tag{S1-9}$$

$$\omega = -\frac{16}{d^2} r r_t' \tag{S1-10}$$

After the substitution of equations (S1-4), (S1-5), (S1-9) and (S1-10) into equation (S1-6), the latter could be written in the following form:

$$-\frac{2}{15} m_0 d^2 \left[\left(\frac{2r}{d}\right)^7\right]''_{tt} = \left[k\pi r^2 \tau - \sigma\pi d\left(1 - \frac{d}{2r}\right)\right] r \tag{S1-11}$$

By multiplying both sides of equation (S1-11) by $2/\sigma\pi d^2$and performing algebraic transformations, one can obtain:

$$\frac{4m_0}{15\sigma\pi}\left[\left(\frac{2r}{d}\right)^7\right]''_{tt} = -\frac{k\tau d}{4\sigma}\left(\frac{2r}{d}\right)^3 + \left(\frac{2r}{d}\right) - 1 \tag{S1-12}$$

Using designation $q = (2r/d)^7$, the equation of motion (S1-12) can be finally written in the dimensionless form:

$$q''_{\tilde{t}\tilde{t}} = -\tilde{\tau} q^{3/7} + q^{1/7} - 1 \tag{S1-13}$$

where $\tilde{t} = t\sqrt{15\sigma\pi/4m_0}$ is proportional to physical time and $\tilde{\tau} = k\tau d/4\sigma$ is proportional to shear stress. The dynamical variable $q(\tilde{t})$ can vary in range from 0 to q_m. $q = 0$ and $q = q_m$ correspond to the completely unfolded and folded state, respectively. The maximum value q_m is found from equation (S1-1) for $x = 0$ and $r = R$:

$$q_m = \left(\frac{3}{2}N\right)^{7/3} \tag{S1-14}$$

One could receive the following relation between the order parameter $u = x/L$ and the variable q: $u = 1 - (q/q_m)^{3/7}$. Obviously, $u = 0$ if the VWF molecule is in its folded state, and $u = 1$ if VWF is in a totally unfolded state.

Equation (S1-13) can be formally considered as a motion equation of a material point having a unit mass and coordinate q in a potential force field:

$$\widetilde{U}_{\tilde{\tau}}(q) = \frac{7}{10}\tilde{\tau}q^{10/7} - \frac{7}{8}q^{8/7} + q \qquad \text{(S1-15)}$$

S1 References

S1.1. Siediecki CA, Lestini BJ, Kottke-Marchant KK, Eppell SJ, Wilson DL, Marchant RE. Shear-dependent changes in the three-dimensional structure of human von Willebrand factor. Blood. 1996;88(8):2939-50.

S1.2. Schneider SW, Nuschele S, Wixforth A, Gorzelanny C, Alexander-Katz A, Netz RR, et al. Shear-induced unfolding triggers adhesion of von Willebrand factor fibers. Proc. Natl. Acad. Sci. USA. 2007;104(19):7899-903.

S1.3. Zlobina KE, Guria GT. Platelet activation risk index as a prognostic thrombosis indicator. Sci. Rep. 2016;6:30508. doi: 10.1038/srep30508

S1.4. De Gennes PG. Kinetics of collapse for a flexible coil. J. Physique Lett. 1985;46(14):639-42.

S1.5. Buguin A, Brochard-Wyart F. Unwinding of globular polymers under strong flows. Macromolecules. 1996;29(14):4937-43.
