# Supplementary material for: Platelet activation via dynamic conformational changes of von Willebrand factor under shear
Source: PLoS One. 2020 Jun 11;15(6):e0234501. doi: 10.1371/journal.pone.0234501 (PMC7289367; doi:10.1371/journal.pone.0234501)
Supplement: S2 Text — (PDF) [file pone.0234501.s002.pdf]

S2 Text. Analysis of solution behaviour at physical boundaries.

According to the obtained in S1 Text expression (S1-15) for the potential energy $\widetilde{U}_{\tilde{\tau}}(q)$, the local minimum of the potential energy (where $q \neq 0$) shifts towards an increase in the variable q with a decrease in the shear stress. When the shear stress is equal to $\tilde{\tau}_*$, the potential energy minimum and extremum are achieved at $q = q_m$ (right boundary of the physical region $[0, q_m]$). Under $\tilde{\tau} < \tilde{\tau}_*$, the extremum of the potential energy is achieved outside the physical region, and the minimum of potential energy is reached at the right boundary of the physical region.

Topological transformations of phase portraits under variations of shear stress are shown in S2-1 Fig.

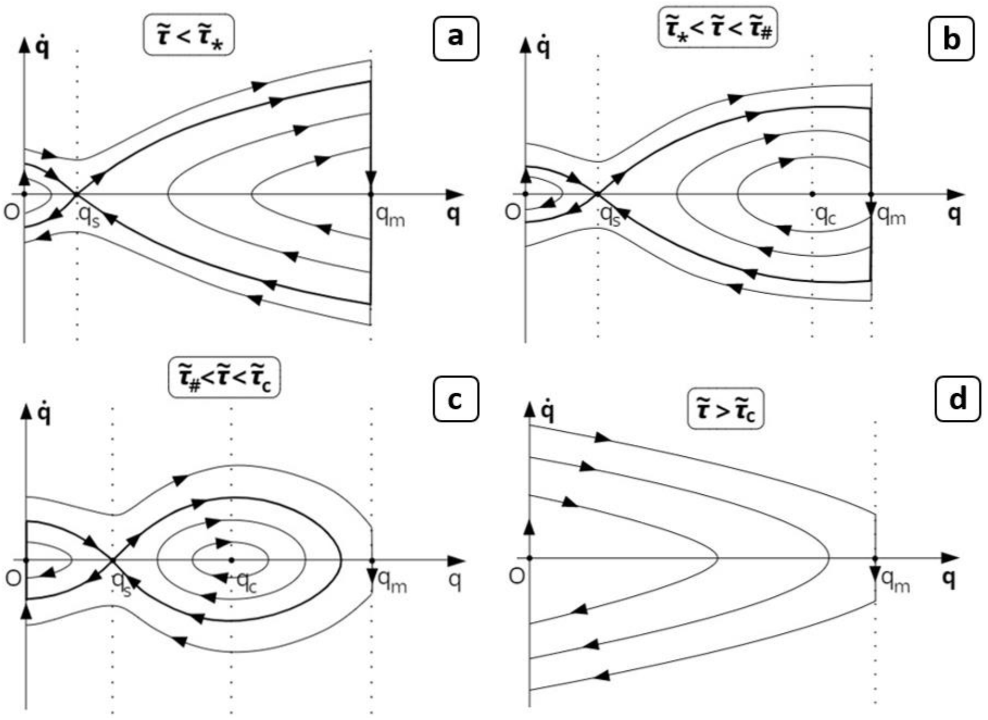

S2-1 Fig. Schematic view of the phase portraits at different values of shear stress, $\tilde{\tau}$. The value $q = 0$ corresponds to the totally unfolded state of the VWF molecule, and $q = q_m$ corresponds to the folded state. Points $q = q_s$ and $q = q_c$ are the maximum and minimum points of the potential energy, $\widetilde{U}_{\tilde{\tau}}(q)$, respectively. Phase portraits under shear stresses (a) $\tilde{\tau} < \tilde{\tau}_*$, (b) $\tilde{\tau}_* < \tilde{\tau} < \tilde{\tau}_\#$, (c) $\tilde{\tau}_\# < \tilde{\tau} < \tilde{\tau}_c$, (d) $\tilde{\tau} > \tilde{\tau}_c$ are shown. The point q_c reaches the margin of the physically allowed region at the point q_m under the shear stress $\tilde{\tau} = \tilde{\tau}_*$.

The conformational state of VWF at time point t is characterized by the value of dynamical variable $q(\tilde{t})$. In this paper, we only analyse the behaviour of solutions of equation (S1-13) in S1 Text under the initial conditions $(q_m; 0)$ (S2-2 Fig, point D). These initial conditions mean that VWF is located on the surface of platelets in the most compact form with a zero value of momentum.

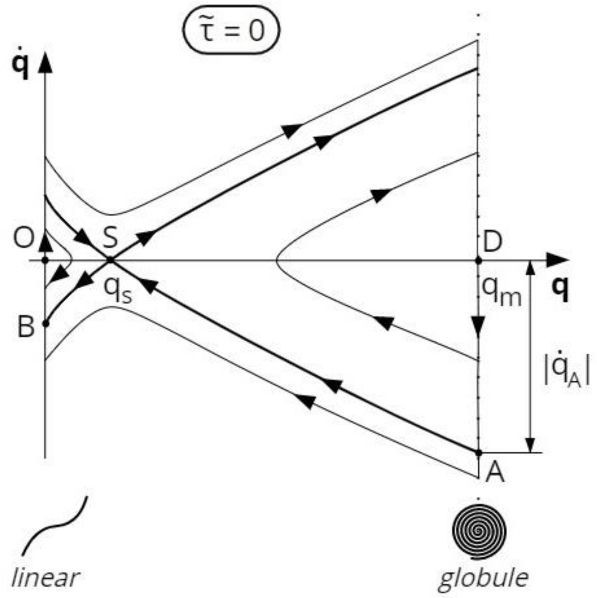

S2-2 Fig. Schematic view of the phase portrait at $\tilde{\tau} = 0$. The value $q = 0$ corresponds to the unfolded state of the VWF molecule; and $q = q_m$, to the folded state. $q = q_s$ is a saddle point, and the curve ASB denotes the separatrix. $|\dot{q}_A|$ is the absolute value of the momentum at point A.

One can see from the phase portrait of the system that the image point should cross the branch AS of the separatrix for total unfolding (S2-2 Fig). It is also clear that the completely unfolded state is always reached with a non-zero value of momentum $|\dot{q}| > |\dot{q}_B| \neq 0$. At the moment of reaching a physical boundary $q = 0$ or $q = q_m$, collision with the boundary should occur.

Denotes by $\tilde{t}_b$ the time moment when the molecule reaches any physical boundary $q = 0$ or $q = q_m$. The momentum value after the collision can be calculated by the following formula:

$$\dot{q}(\tilde{t}_b + \Delta\tilde{t}) = -\alpha\dot{q}(\tilde{t}_b) \quad \text{(S2-1)}$$

where $\dot{q}(\tilde{t}_b)$ and $\dot{q}(\tilde{t}_b + \Delta\tilde{t})$ are values of momentum just before and after collision, respectively, and α is the restitution coefficient characterizing the physical boundary ($\alpha \in [0; 1]$) [S2.1]. Equation (S2-1) will be used below for the analysis of solution behaviour.

For the sake of simplicity, let us first suppose that high shear stress varies over time as a delta function. Moreover, suppose that such shear impulse imparts momentum to VWF that is sufficient for crossing part AS of the separatrix. Then, after the action of the shear impulse, the image point will be somewhere below the point $A(q_m; \dot{q}_A)$ (S2-2 Fig). Moving along the corresponding trajectory, the image point reaches the left boundary of the physical domain somewhere under point $B(0; \dot{q}_B)$. The further behaviour of the solution depends on the value of α (equation (S2-1)).

If $\alpha = 1$, absolutely elastic collision with the boundary will occur. In this case, the solution is periodic, and the VWF molecule moves from the folded to the totally unfolded state and back (S2-3a Fig). If the condition $0 < \alpha < 1$ is fulfilled, partially elastic collision occurs. Partial loss of the momentum will take place at every collision. Depending on the value of α, the image point will finally be in one of the boundary states $O(0; 0)$ or $D(q_m; 0)$ (S2-3b and S2-3c Figs). Finally, in the case of absolutely inelastic collision ($\alpha = 0$), the image point will move into a totally unfolded state $O(0; 0)$ after the first collision with the boundary $q = 0$ (S2-3d Fig). It is worth mentioning that the behaviour of the solution will be qualitatively similar for any profile of shear impulse whose strength is sufficient for crossing branch AS of the separatrix.

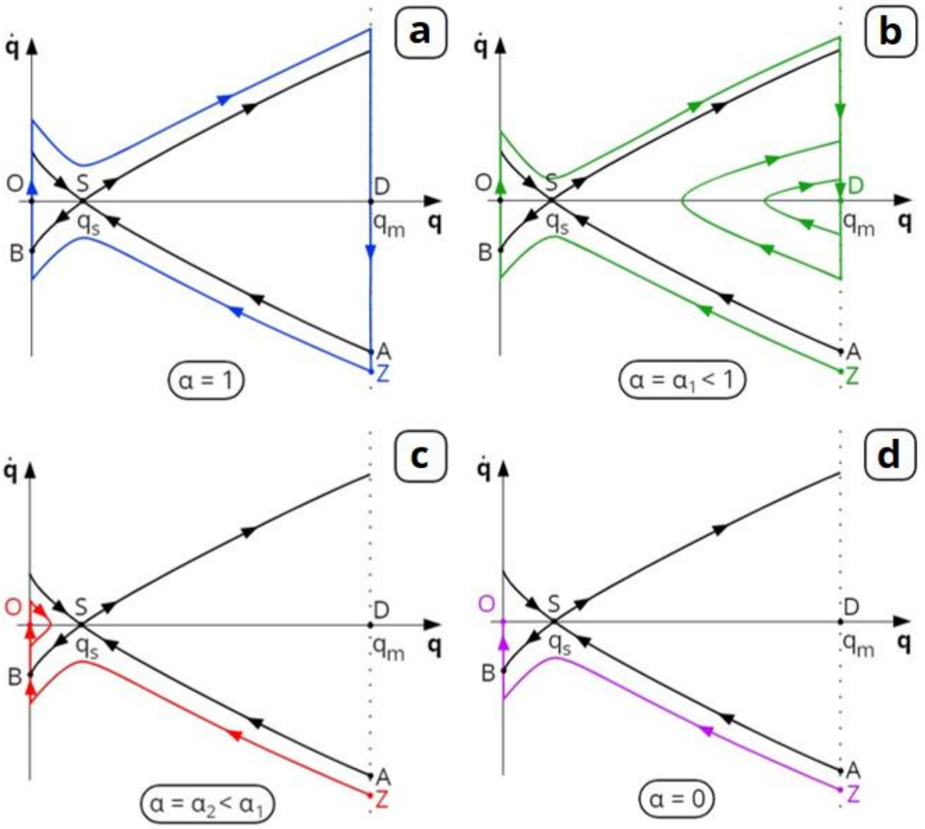

S2-3 Fig. Dynamics of VWF molecule unfolding at different values of the restitution coefficient α at the fixed value of initial momentum. Point Z indicates the state of the system after the instantaneous overcritical action of a shear stress impulse ($|\dot{q}_Z| > |\dot{q}_A|$). There are several variants of the molecular behaviour depending on the value of the coefficient α. (a) Molecule periodically oscillates on the surface of platelet ($\alpha = 1$, blue trajectory). (b) Trajectory gets into the attraction zone of the centre, and the molecule gradually moves to a totally folded state $(q_m, 0)$ ($\alpha = \alpha_1 < 1$, green trajectory). (c) Trajectory falls into the attraction zone of a totally unfolded state $O(0,0)$ after the first collision with a boundary ($\alpha = \alpha_2 < \alpha_1$, red trajectory). (d) Molecule loses all its momentum at the first collision and remains in a totally unfolded state $O(0,0)$.

In the current work, we suppose that the value of the coefficient α is sufficiently small, so after the first collision with boundary $q = 0$, the image point will always fall into the attraction zone of the fully unfolded state $O(0; 0)$.

It is worth to note that equation of motion (S1-13) in S1 Text does not imply that any dissipative effects are involved. The schematic view of the phase portrait for the case of small

dissipation is presented in S2-4 Fig. The account of small dissipation does not lead to changes in critical point locations. However, the critical value of momentum that is sufficient for total VWF unfolding becomes larger than in the case without dissipation.

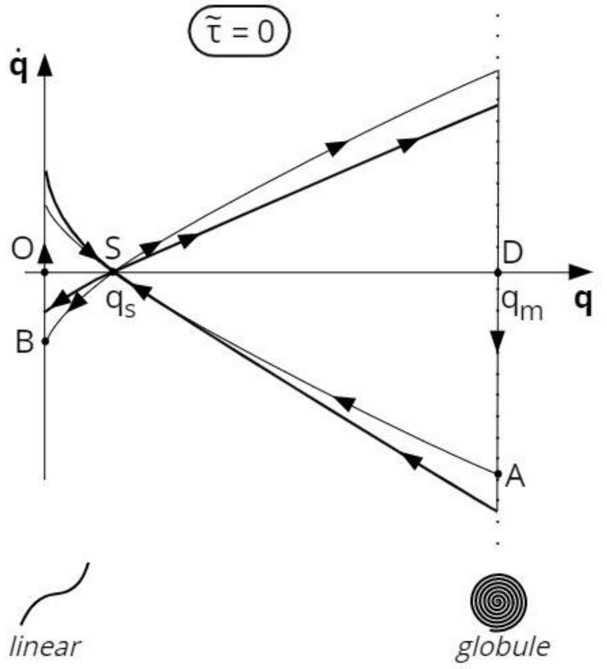

S2-4 Fig. The transformation of the system phase portrait due to small dissipation. The bold and normal black lines correspond to the separatrix in the presence and absence of dissipation, respectively.

S2 References

S2.1. Tabor D. A simple theory of static and dynamic hardness. Proc. R. Soc. Lond. A. Math. Phys. Sci. 1948;192(1029):247-74.
