# Supplementary material for: Platelet activation via dynamic conformational changes of von Willebrand factor under shear
Source: PLoS One. 2020 Jun 11;15(6):e0234501. doi: 10.1371/journal.pone.0234501 (PMC7289367; doi:10.1371/journal.pone.0234501)
Supplement: S3 Text — (PDF) [file pone.0234501.s003.pdf]

S3 Text. Derivation of the critical condition of the total VWF unfolding.

Consider a VWF molecule passing through a high shear stress zone. $\dot{q}_{in}$ and $\dot{q}_{out}$ correspond to momentum at moments $\tilde{t}_{in}$ and $\tilde{t}_{out}$, respectively, when platelets go in and out of the high shear stress zone. Suppose that the VWF molecule is in a folded state at the initial moment $\tilde{t}_{in}$. That formally means that $q(\tilde{t}_{in}) = q_m$ and $\dot{q}_{in} \equiv \dot{q}(\tilde{t}_{in}) = 0$. Looking at the phase portrait (S2-2 Fig in S2 Text), it becomes clear that the sufficient condition of total VWF unfolding has the form:

$$|\dot{q}_{out}| > |\dot{q}_A| \qquad \text{(S3-1)}$$

First, we find the value of $\dot{q}_A$. Considering that the dissipation of energy is not explicitly taken into account in the current approach, one of the first integrals has the following form:

$$\widetilde{U}_{\tilde{\tau}}(q) + \frac{\dot{q}^2}{2} = const \qquad \text{(S3-2)}$$

In particular, it may be applied to the branch of the separatrix AS (S2-2 Fig in S2 Text). Taking into account that the value of kinetic energy at point S is equal to zero, one can obtain:

$$\widetilde{U}_0(q_m) + \frac{\dot{q}_A^2}{2} = \widetilde{U}_0(q_s) \qquad \text{(S3-3)}$$

Therefore, for the value of the momentum at point A, it is easy to obtain:

$$|\dot{q}_A| = \sqrt{2\left(\widetilde{U}_0(q_s) - \widetilde{U}_0(q_m)\right)} \qquad \text{(S3-4)}$$

Based on equation (S1-13) in S1 Text, it is possible to find the momentum value $\dot{q}_{out}$, that a VWF molecule obtained when passing through the high shear stress zone. In fact, the value of momentum change can be found by integrating equation (S1-13) in S1 Text with respect to time:

$$\dot{q}_{out} - \dot{q}_{in} = \int_{\tilde{t}_{in}}^{\tilde{t}_{out}} \ddot{q}\, d\,\tilde{t} = \int_{\tilde{t}_{in}}^{\tilde{t}_{out}} \left(-\tilde{\tau} q^{\frac{3}{7}} + q^{\frac{1}{7}} - 1\right) d\,\tilde{t} \qquad \text{(S3-5)}$$

Taking into account that $\dot{q}_{in} = 0$, equation (S3-5) can be transformed into the following form:

$$|\dot{q}_{out}| = \int_{\tilde{t}_{in}}^{\tilde{t}_{out}} q^{\frac{3}{7}}\tilde{\tau}(\tilde{t})d\tilde{t} - \int_{\tilde{t}_{in}}^{\tilde{t}_{out}} \left(q^{\frac{1}{7}} - 1\right) d\tilde{t} \tag{S3-6}$$

By substituting equations (S3-4) and (S3-6) into inequality (S3-1), the necessary condition for complete VWF unfolding takes the form:

$$\begin{gathered} |\dot{q}_{out}| = \int_{\tilde{t}_{in}}^{\tilde{t}_{out}} q^{\frac{3}{7}}\tilde{\tau}(\tilde{t})d\tilde{t} - \int_{\tilde{t}_{in}}^{\tilde{t}_{out}} \left(q^{\frac{1}{7}} - 1\right) d\tilde{t} > \\ > \sqrt{2\left(\widetilde{U}_0(q_s) - \widetilde{U}_0(q_m)\right)} = |\dot{q}_A| \end{gathered} \tag{S3-7}$$

Equation (S3-7) could be transformed to the following form:

$$\int_{\tilde{t}_{in}}^{\tilde{t}_{out}} q^{\frac{3}{7}}\tilde{\tau}(\tilde{t})d\tilde{t} > \sqrt{2\left(\widetilde{U}_0(q_s) - \widetilde{U}_0(q_m)\right)} + \int_{\tilde{t}_{in}}^{\tilde{t}_{out}} \left(q^{\frac{1}{7}} - 1\right) d\tilde{t} \tag{S3-8}$$

Keeping in mind that $q > q_s = 1$ under $\tilde{\tau} = 0$, one can find that $q^{1/7} - 1 > 0$, and consequently, $\int_{\tilde{t}_{in}}^{\tilde{t}_{out}} (q^{1/7} - 1) d\tilde{t} > 0$. Thus, the following inequality is satisfied:

$$\int_{\tilde{t}_{in}}^{\tilde{t}_{out}} q^{\frac{3}{7}}\tilde{\tau}(\tilde{t})d\tilde{t} > \sqrt{2\left(\widetilde{U}_0(q_s) - \widetilde{U}_0(q_m)\right)} \tag{S3-9}$$

At the same time, it is obvious that the values of the dynamical variable $q(t)$ during VWF unfolding are always less than q_m ($q(t) < q_m$). Therefore, the following inequality is valid:

$$q_m^{\frac{3}{7}} \int_{\tilde{t}_{in}}^{\tilde{t}_{out}} \tilde{\tau}(\tilde{t})d\tilde{t} > \int_{\tilde{t}_{in}}^{\tilde{t}_{out}} q^{\frac{3}{7}}\tilde{\tau}(\tilde{t})d\tilde{t} \tag{S3-10}$$

By comparing the expressions (S3-9) and (S3-10), one can obtain:

$$CSS = \int_{\tilde{t}_{in}}^{\tilde{t}_{out}} \tilde{\tau}(\tilde{t})d\tilde{t} > \frac{1}{q_m^{\frac{3}{7}}} \sqrt{2\left(\widetilde{U}_0(q_s) - \widetilde{U}_0(q_m)\right)} \equiv CSS_0 \tag{S3-11}$$

where CSS is the cumulative shear stress and CSS_0 is its critical value. Expression (S3-11) represents the necessary but not sufficient condition of VWF unfolding on the surface of platelets. In other words, to unfold a VWF multimer, the value of the cumulative shear stress must be at least larger than the right-hand side of equation (S3-11).

It is worth noting that the developed approach gives us the opportunity not only to find an expression for the condition of the cumulative shear stress (equation (S3-11)) but also to find the dependence of CSS_0 as a function of N. Indeed, taking into consideration that $q_s = 1,\ \ q_m = (3N/2)^{7/3}$ and that the expression for potential energy has a form $\widetilde{U}_0(q) = -(7/8)q^{8/7} + q$, the value of the critical cumulative shear stress (CSS_0) is given by the following formula:

$$CSS_0 = \left(\frac{3}{2}N\right)^{\frac{1}{3}} \sqrt{\frac{7}{4} + \frac{1}{4}\left(\frac{3}{2}N\right)^{-\frac{8}{3}} - 2\left(\frac{3}{2}N\right)^{-\frac{1}{3}}} \qquad \text{(S3-12)}$$

Equation (S3-12) represents an explicit expression for the dependence of the critical cumulative shear stress CSS_0 value on the multimeric size of VWF molecules. Thus, the value of the critical cumulative shear stress is an increasing function of the VWF multimer size N.
