# Supplementary material for: Platelet activation via dynamic conformational changes of von Willebrand factor under shear
Source: PLoS One. 2020 Jun 11;15(6):e0234501. doi: 10.1371/journal.pone.0234501 (PMC7289367; doi:10.1371/journal.pone.0234501)
Supplement: S4 Text — (PDF) [file pone.0234501.s004.pdf]

S4 Text. VWF unwinding under the action of rectangular shear stress profile.

The unfolding of the VWF molecule under the action of the rectangular shear stress profile was investigated by the numerical solution to equation (S1-13) in S1 Text (S4-1 Fig). At a fixed value of shear profile amplitude $\tilde{\tau}_m$, the molecule will unwind to its full length if the duration of impulse $\tilde{t}_G$ will be longer than the time $\tilde{t}_F$ during which the trajectory reaches part AS of the separatrix. The goal of numerical experiments was to build the dependence $\tilde{t}_F(\tilde{\tau}_m)$.

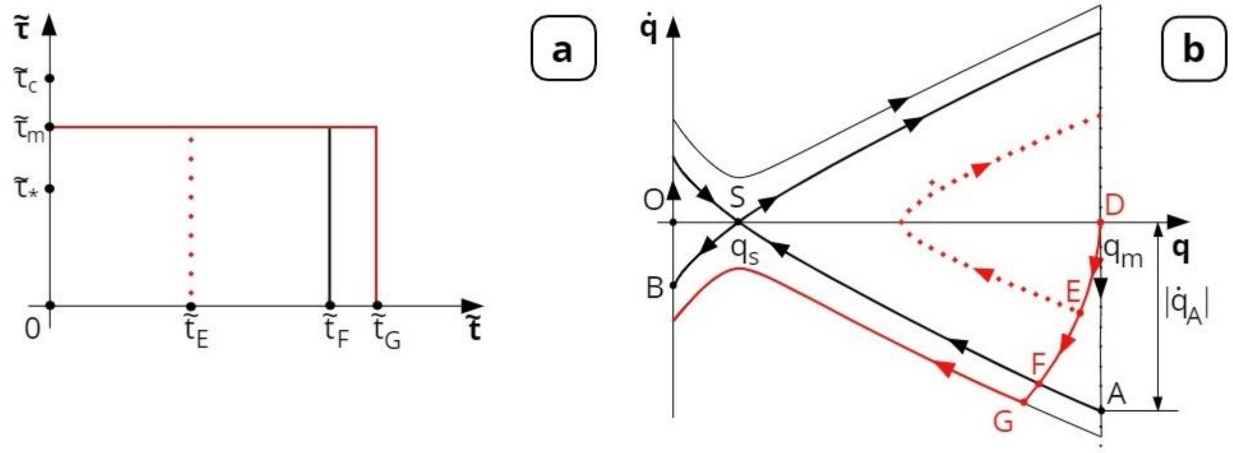

S4-1 Fig. VWF molecule unfolding under the action of a rectangular shear stress profile with various durations. (a) Shear stress impulse $\tilde{\tau}(\tilde{t})$ is shown in red, $\tilde{\tau}_m$ is its amplitude, and $\tilde{t}_G$ is its duration. (b) Phase trajectory of the solution (equation (S1-13) in S1 Text) is illustrated by the red line for the shear stress profile from S3-1a Fig in S3 Text; initial conditions: $q = q_m$, $\dot{q} = 0$. $\tilde{t}_E$, $\tilde{t}_F$ and $\tilde{t}_G$ are time moments when the image point reaches points E, F and G, respectively. If the duration of the shear stress impulse $\tilde{t}_G$ is greater than that of $\tilde{t}_F$, the trajectory crosses the part of the separatrix AS, and the molecule can unwind to its full length. $|\dot{q}_A|$ is the absolute value of the momentum at point A.

Equation (S1-13) in S1 Text was solved under the initial conditions $(q_m; 0)$ (point D in S4-1 Fig). The time point when trajectory crossed the branch AS of the separatrix was chosen as $\tilde{t}_F$. The equation of the branch AS of the separatrix derived by the traditional procedure of analytical mechanics [S4.1] was given by the following equation:

$$\dot{q} = -\sqrt{-\frac{7}{4}\left(q_s^{8/7} - q^{8/7}\right) + 2(q_s - q)} \tag{S4-1}$$

The numerical integration of equation (S1-13) in S1 Text was carried out by BS23 algorithm [S4.2] using custom-written MATLAB (license number #1099706, version R2016b, The MathWorks) scripts.

The results of numerical experiments for fixed value of multimer size N are shown in S4-2 Fig. The curve $\tilde{t}_F(\tilde{\tau}_m)$ has a vertical asymptote at the value $\tilde{\tau}_m = \tilde{\tau}_\#$ (vertical dotted line in S4-2 Fig). The value $\tilde{\tau}_\#$ is calculated by numerical solving of the equation:

$$\widetilde{U}_{\tilde{\tau}_\#}(q_s) = \widetilde{U}_{\tilde{\tau}_\#}(q_m) \quad \text{(S4-2)}$$

where $\widetilde{U}_{\tilde{\tau}_\#}(q)$ is the potential energy of the system at $\tilde{\tau} = \tilde{\tau}_\#$ (equation (S3-15) in S3 Text). The dependence of $\tilde{\tau}_\#$ as a function of N is shown in S4-3 Fig. It is observed that $\tilde{\tau}_\#$ decreases with increasing number of multimers in the VWF molecule and that the following condition is satisfied: $\tilde{\tau}_\# \leq \tilde{\tau}_c \equiv 4/27$. With increasing amplitude of the shear impulse, the dependence $\tilde{t}_F(\tilde{\tau}_m)$ asymptotically tends to the following curve (dashed line in S4-2 Fig):

$$\tilde{\tau}_m \tilde{t}_F = CSS_0 \quad \text{(S4-3)}$$

where CSS_0 is a value of critical cumulative shear stress calculated by expression (S3-12) in S3 Text.

The value of CSS_0 is related to the critical momentum value $\dot{q}_A$ (equations (S3-7) and (S3-11) in S3 Text) in the following way:

$$\dot{q}_A = q_m^{3/7} CSS_0 \quad \text{(S4-4)}$$

Additionally, the characteristic unfolding time $\tilde{t}_0$ may be calculated as

$$\tilde{t}_0 = \frac{q_m}{\dot{q}_A} \quad \text{(S4-5)}$$

The $\tilde{t}_F$ was measured in units of $\tilde{t}_0$ in S4-2 Fig.

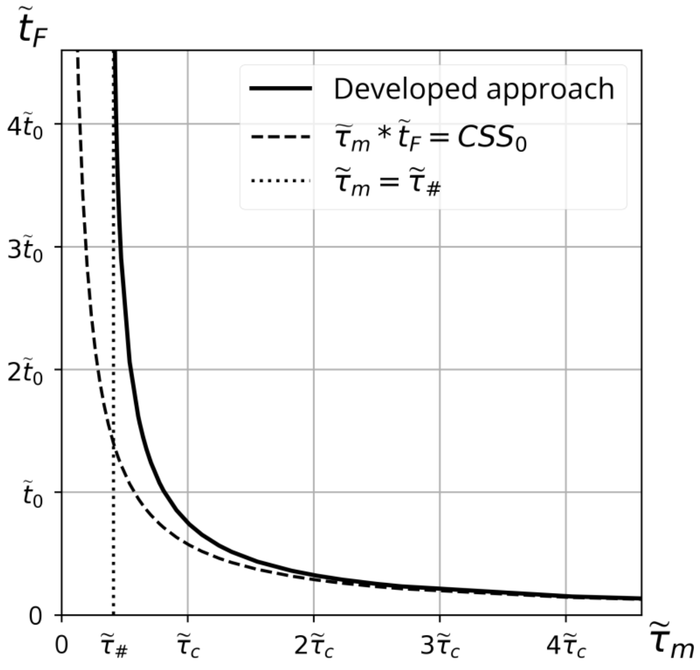

S4-2 Fig. Dependence of critical time duration $\tilde{t}_F$ on amplitude $\tilde{\tau}_m$ of a rectangular shear stress impulse. The solid curve corresponds to the dependence $\tilde{t}_F(\tilde{\tau}_m)$ obtained in the framework of developed approach. The dotted line $\tilde{\tau}_m = \tilde{\tau}_\#$ is a vertical asymptote of the curve $\tilde{t}_F(\tilde{\tau}_m)$. The curve $\tilde{t}_F(\tilde{\tau}_m)$ is asymptotically tending to the dashed curve with increasing $\tilde{\tau}_m$. The values of $\tilde{t}_F$ are measured in units of characteristic unfolding time $\tilde{t}_0$ (equation (S4-5)). The value of CSS_0 is given by equation (S3-12) in S3 Text. All curves were built for the case $N = 36$.

S4-4 Fig shows the dependencies $\tilde{t}_F(\tilde{\tau}_m)$ for two different values of N. As one can see, two dependencies demonstrate qualitatively similar behaviour.

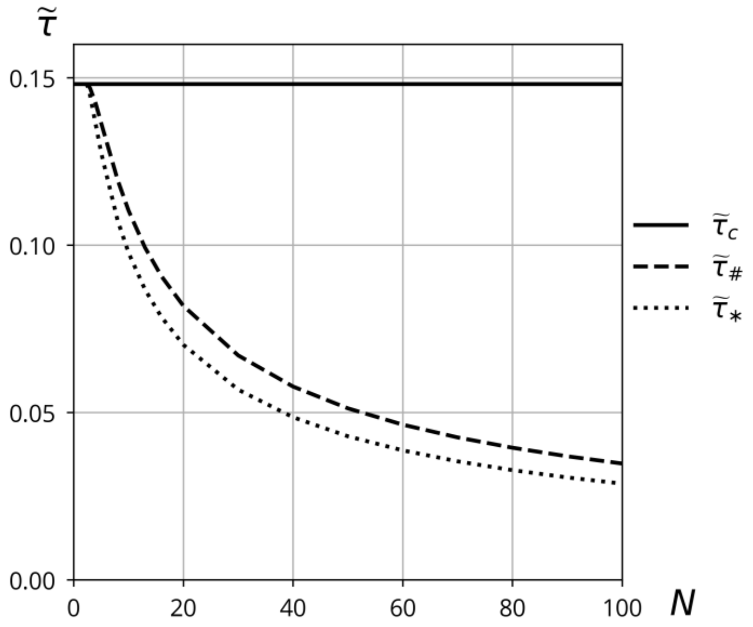

S4-3 Fig. The dependence of critical shear stress values $\tilde{\tau}_*$ and $\tilde{\tau}_\#$ on VWF multimer size N. $\tilde{\tau}_*$ is a value of shear stress above which the VWF molecule begins to unwind. $\tilde{\tau}_\#$ is a value of shear stress above which the VWF molecule can unwind on full length. The dependence $\tilde{\tau}_\#(N)$ was built numerically via solution of equation (S4-2). The value $\tilde{\tau}_c$ is a value of shear stress under which VWF molecules can unwind on full length regardless of its size. The dependencies $\tilde{\tau}_*$ and $\tilde{\tau}_c$ were obtained in the work [S4.3].

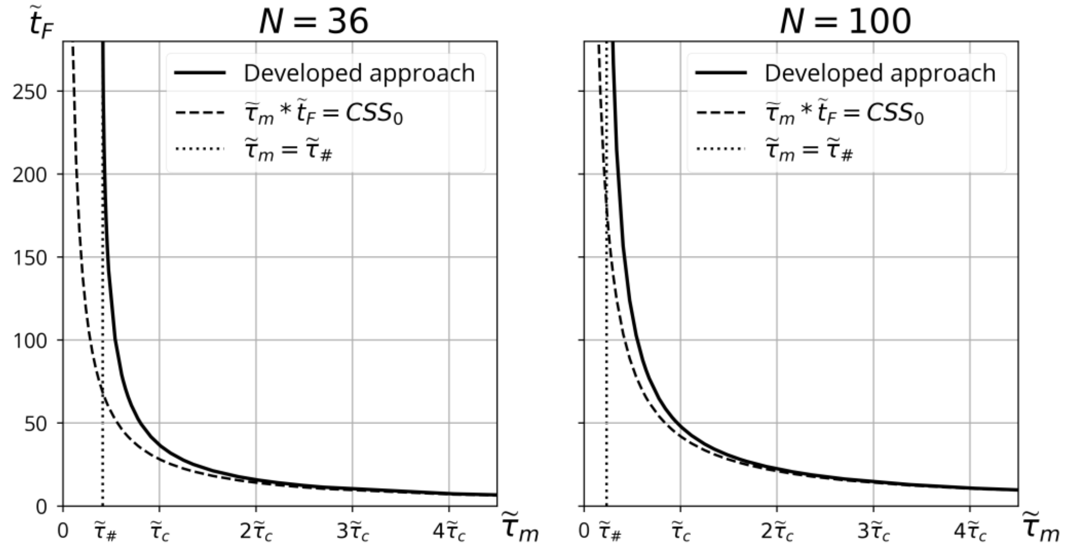

S4-4 Fig. Dependence of critical time $\tilde{t}_F$ от $\tilde{\tau}_m$ for two different values of N. Solid lines represent dependencies $\tilde{t}_F(\tilde{\tau}_m)$ obtained in the framework of developed approach, while dashed lines display dependencies, calculated via equation (S4-3).

It follows from S4-3 Fig that the value of the shear stress threshold ($\tau_{\#}$) asymptotically depends on the VWF multimer size as a power law ($\tau_{\#} \sim N^{-2/3}$). This means that the level of SIPAct in blood-wetted devices as well as in stenotic vessels may be directly regulated by the variation of VWF multimer size.

S4 References

S4.1. Andronov AA, Vitt AA, Khaikin SE. Non-linear conservative systems. In: Fishwick W, editor. Theory of oscillators. Pergamon Press; 1966. pp. 74-145.

S4.2. Bogacki P, Shampine LF. A 3 (2) pair of Runge-Kutta formulas. Appl. Math. Lett. 1989;2(4):321-5.

S4.3. Zlobina KE, Guria GT. Platelet activation risk index as a prognostic thrombosis indicator. Sci. Rep. 2016;6:30508. doi: 10.1038/srep30508
